# Microbe–disease associations prediction by graph regularized non‐negative matrix factorization with $L_{2,1}$ norm regularization terms

**DOI:** 10.1111/jcmm.18553

**Published:** 2024-09-06

**Authors:** Ziwei Chen, Liangzhe Zhang, Jingyi Li, Hang Chen

**Affiliations:** ^1^ School of Electronic and Information Engineering Beijing Jiaotong University Beijing China

**Keywords:** graph dual regularization $L_{2,1}$ norm regularization, inertial proximal alternating linearized minimization, microbe–disease association, non‐negative matrix factorization

## Abstract

Microbes are involved in a wide range of biological processes and are closely associated with disease. Inferring potential disease‐associated microbes as the biomarkers or drug targets may help prevent, diagnose and treat complex human diseases. However, biological experiments are time‐consuming and expensive. In this study, we introduced a new method called iPALM‐GLMF, which modelled microbe–disease association prediction as a problem of non‐negative matrix factorization with graph dual regularization terms and $L_{2,1}$ norm regularization terms. The graph dual regularization terms were used to capture potential features in the microbe and disease space, and the $L_{2,1}$ norm regularization terms were used to ensure the sparsity of the feature matrices obtained from the non‐negative matrix factorization and to improve the interpretability. To solve the model, iPALM‐GLMF used a non‐negative double singular value decomposition to initialize the matrix factorization and adopted an inertial Proximal Alternating Linear Minimization iterative process to obtain the final matrix factorization results. As a result, iPALM‐GLMF performed better than other existing methods in leave‐one‐out cross‐validation and fivefold cross‐validation. In addition, case studies of different diseases demonstrated that iPALM‐GLMF could effectively predict potential microbial‐disease associations. iPALM‐GLMF is publicly available at https://github.com/LiangzheZhang/iPALM‐GLMF.

## 1 INTRODUCTION

Microbes are a diverse group of microscopic organisms existing in either single‐cell or multicellular forms, primarily categorized into bacteria, fungi, viruses, archaea and protozoa.^1^ They are a ubiquitous and important part of all ecosystems, with their habitats ranging widely, extending even to harsh environments such as polar regions and the ocean depths.^2^ Therefore, it is not surprising to find a wealth of symbiotic microbes thriving in the human body, including on the skin, in the gut and throughout the oral cavity.^3^ Research indicates that they play significant roles in human health and disease, such as maintaining internal equilibrium,^4^ developing the immune system^5^ and resisting pathogens.^2^ For instance, studies have indicated that the proliferation of pathogenic bacteria in the oral cavity may lead to an inflammatory disease known as periodontitis.^6^ The findings demonstrate that periodontitis‐associated microbial communities have highly conserved changes in metabolic and virulence gene expression profiles, whereas healthy samples did not. This suggests that changes in the composition of the oral microbial community may be related to the pathogenesis of periodontitis.^7^ Furthermore, there is clinical and histological evidence that topical application of lactic acid can be effective in depigmenting the skin, improving the surface roughness of the skin and reducing mild wrinkles caused by environmental photodamage.^8^ Consequently, elucidating the relationship between human diseases and microbes can not only enhance our comprehension of disease pathogenesis, but also provide novel strategies for disease diagnosis and treatment.^9^, ^10^, ^11^, ^12^, ^13^ Unfortunately, reliance on traditional experimental methods is both laborious and time‐consuming, and it is challenging to fully explore potential microbe–disease associations (MDAs) within a limited timeframe. Consequently, there has been a growing interest in computational models that can predict disease‐associated microbes.^14^

In recent years, numerous computational models, including those based on scoring functions, have been developed to predict potential microbe–disease associations. For instance, Chen et al.^15^ proposed the first computational model in this field called KATZHMDA, which is based on the KATZ method. In this model, the prediction of potential associations is transformed into an integration based on the number of walks in the network and its own length. It is a valid metric for calculating the probability of potential associations between microbes and diseases. Huang et al.^16^ presented the computational model of Path‐Based Human Microbe‐Disease Associations prediction (PBHMDA), which is based on a depth‐first search algorithm for predicting microbes that may be associated with diseases. The model generates a prediction score for each microbe–disease association pair by constructing a heterogeneous network and traversing all connection paths between nodes in the heterogeneous network using a specialized depth‐first search algorithm. Long and Luo^17^ proposed a novel computational model for Weighted Meta‐Graph‐based Human Microbe‐Disease Associations prediction (WMGHMDA). The model iteratively implements a pre‐designed weighted meta‐graph search algorithm on a heterogeneous information network, and discovers possible microbe–disease pairs by accumulating the contribution value of the weighted meta‐graph to the microbe–disease pairs as a probability score. Xu et al.^18^ developed a novel computational method to discover potential Microbe‐Disease Associations based on the Kronecker Regularized Least Squares (MDAKRLS). The model is designed with Kronecker regularized least squares that have different Kronecker similarities to obtain the prediction scores separately, and the final prediction scores are calculated by integrating the contributions of different similarities. The advantages of these models are that the theory of the algorithms and computational processes involved is relatively easy to understand and the models do not require negative samples for prediction, while the disadvantage is that most models based on scoring functions are not applicable to new diseases.

Researchers have also developed network models for microbe–disease associations prediction. For example, Bao et al.^19^ proposed the model of Network Consistency Projection for Human Microbe‐Disease Associations prediction (NCPHMDA), where the model constructs the similarity of nodes in a heterogeneous network to measure the correlation between microbes and diseases, and calculates the consistency projection score to infer latent microbes for diseases. Huang et al.^20^ constructed a new model to reveal potential microbial–disease associations by integrating two independent recommendation models: a neighbour‐based prediction model and a graph‐based prediction model. Wu et al.^21^ presented a novel computational model employing Random Walking with Restart optimized by Particle Swarm Optimization (PSO) on the heterogeneous interlinked network of Human Microbe‐Disease Associations (PRWHMDA). The model optimizes the random walk and restart of a heterogeneous network of human microbe–disease associations, using a PSO to optimize the random walk parameters and obtain the final association probability vector. Yan et al.^22^ proposed a correlation prediction method (BRWMDA) based on similarity and improving bi‐random walk on the disease and microbe networks. The method utilizes network integration and double random walks on the disease and microbe networks. When the maximum number of iterations of both networks is reached, the random walk stops and produces the final correlation probability matrix. The main advantage of these models is that they can fully utilize the topological information in the network. In addition, these models involve fewer parameters, which greatly reduces the difficulty of parameter selection, but the disadvantage is that some network‐based approaches rely heavily on experimentally validated microbe–disease associations, and cannot predict new diseases or microbes in the absence of known association information.

Machine learning techniques especially deep learning have obtained wide applications in bioinformatics due to their better classification performance.^23^, ^24^, ^25^, ^26^, ^27^, ^28^ Concurrently, many computational methods have been developed to help identify the relationship between microbes and diseases. For example, Peng et al.^29^ developed a model of Adaptive Boosting for Human Microbe‐Disease Associations prediction (ABHMDA), which reveals microbes associated with a disease by a strong classifier consisting of weak classifiers with their own weights. ABHMDA assigns different weights to multiple weak classifiers to get the final association. Wang et al. developed a semi‐supervised computational model of Laplacian Regularized Least Squares for Human Microbe‐Disease Associations (LRLSHMDA) with good results. Li et al.^30^ proposed a novel computational method called BPNNHMDA. The method takes advantage of the fact that the neural network model, including a unique activation function and optimized initial connection weights based on Gaussian interaction profile kernel similarity, which effectively improves the training speed of the model. Hua et al.^31^ developed a model of Multi‐View Graph Convolutional Network for Microbe‐Disease Associations prediction (MVGCNMDA), which employs specific data augmentation and multi‐view attentional blocks to reveal microbes associated with diseases. Chen et al.^32^ proposed an approach to predict MDAs based on heterogeneous networks and metapath aggregation graph neural networks (MATHNMDA). The model utilizes heterogeneous networks as inputs to the metapath aggregation graph neural network, and employs aggregation and attention mechanisms among metapaths to integrate the semantic information of all the different metapaths, thereby obtaining the final embeddings of microbial nodes and disease nodes.

The discovery of potential microbe–disease associations will undoubtedly be of great help in research to understand disease pathogenesis and develop treatments for human diseases. Since traditional biological experiments are generally time‐consuming and labour‐intensive, efficient and reliable computational prediction methods are urgently needed. In recent, great progress has been made in developing computational models for predicting potential microbe–disease associations. As a machine learning method, the matrix factorization approach has proven to be an effective tool and has been widely used in bioinformatics research. For example, Gönen^33^ proposed a kernelized Bayesian matrix factorization with twin kernels method to predict drug‐target interactions. He et al.^34^ developed a novel predictive model of Graph Regularized Non‐negative Matrix Factorization for Human Microbe‐Disease Association prediction (GRNMFHMDA).

In this study, we put forward a novel computational model named iPALM‐GLMF, which was a non‐negative matrix factorization model based on graph dual regularization terms and $L_{2,1}$ norm regularization terms. The graph dual regularization terms were used to integrate the geometric information of the microbe similarity matrix and the disease similarity matrix, and the $L_{2,1}$ norm regularization terms were used to ensure the sparsity of the matrices obtained from the non‐negative matrix factorization. We then used the non‐negative double singular value decomposition (NNDSVD)^35^ to provide valid and interpretable initial component matrices for the matrix factorization and used an inertial proximal alternating linear minimization iterative process, which has been shown to converge to the KKT point, to obtain the final resultant matrix factorization.^36^

Overall, our main contributions were summarized as follows:

- We introduced a novel approach to improve nonnegative matrix factorization by adding graph dual regularization terms and $L_{2,1}$ norm regularization terms.
- By using manifold theory, introducing graph dual regularization terms to efficiently integrate different similarity matrices and preserve manifold features of the data space.
- To improve interpretability and mitigate the effects of inherent noise in the microbe and disease feature spaces, $L_{2,1}$ norm regularization terms was applied to the feature matrices to select the most representative or discriminative sparse features.
- NNDSVD was used to initialize the non‐negative matrix factorization and to solve the matrix factorization using a fast convergent inertial proximal alternating linearization minimization algorithm.

Numerous experimental results have shown that iPALM‐GLMF has better performance than other state‐of‐the‐art methods. Experimental results on two data sets, that is, HMDAD and Disbiome, indicate that iPALM‐GLMF model consistently outperforms the other five state‐of‐the‐art methods. Case studies of three common diseases, colorectal cancer, inflammatory bowel disease (IBD) and asthma, further validate the effectiveness of iPALM‐GLMF.

## 2 MATERIAL

### 2.1 Human microbe–disease associations

To establish the human microbe–disease interaction network, we retrieved known microbe–disease associations from the Human Microbe‐Disease Association Database (HMDAD) (http://www.cuilab.cn/hmdad).^37^ There were 483 experimentally confirmed microbe–disease associations between 39 diseases and 292 microbes. After removing redundant associations, we obtained 450 associations. In addition, Janssens et al. released a new microbe–disease association database called Disbiome (https://disbiome.ugent.be/home), in which 5573 experimentally confirmed human microbe–disease associations were collected from previously published literature and different databases, including 240 diseases and 1098 microbes.^38^ In Disbiome, a microbe–disease pair may be recorded multiple times depending on the assay. After filtering out duplicates, we ended up downloading 4351 associations between 218 diseases and 1052 microbes. Overall, the specific statistics of the two microbe–disease association datasets are shown in Table 1.

**TABLE 1:** The information of two microbe–disease associations datasets.

Dataset	# Microbes	# Diseases	# Association
HMDAD	292	39	450
Disbiome	1052	218	4351

For better description, we formulated microbe–disease associations as a binary matrix $A\in {\mathbb{R}}^{m\times d}$ with m and d representing the numbers of microbes and diseases, respectively. If there exists an experimentally verified relationship between a microbe $m_{i}$ and a disease $d_{j}$, $A_{\mathrm{ij}}$ equals to 1, otherwise 0.

### 2.2 Gaussian interaction profile kernel similarity for diseases and microbes

We calculated Gaussian kernel similarity for microbes and diseases based on the hypothesis that microbes related to common diseases are more likely to show same functions.^39^ Specifically, since the *i*th row and the *j*th column of the adjacency matrix A denote the interactions between microbes $m_{i}$ or disease $d_{j}$ and all microbes or all diseases, we denote $\mathrm{IP}\left(m_{i}\right)$ and $\mathrm{IP}\left(d_{j}\right)$ as the interaction profiles of microbe $m_{i}$ with disease $d_{j}$, respectively. The Gaussian kernel similarity between microbes and diseases are defined as follows:

(1) $$\mathrm{GM}\left(m_{i},m_{j}\right)=\exp\left(-\gamma_{m}{\left\|\mathrm{IP}\left(m_{i}\right)-\mathrm{IP}\left(m_{j}\right)\right\|}^{2}\right),$$

(2) $$\mathrm{GD}\left(d_{i},d_{j}\right)=\exp\left(-\gamma_{d}{\left\|\mathrm{IP}\left(d_{i}\right)-\mathrm{IP}\left(d_{j}\right)\right\|}^{2}\right),$$

where $\gamma_{m}$ and $\gamma_{d}$ represent the normalized kernel bandwidths and are defined as follows:

(3) $$\gamma_{m}=\frac{\gamma_{m}^{\prime }}{\left(\frac{1}{m}\sum_{i=1}^{m}{\left\|\mathrm{IP}\left(m_{i}\right)\right\|}^{2}\right)},$$

(4) $$\gamma_{d}=\frac{\gamma_{d}^{\prime }}{\left(\frac{1}{d}\sum_{i=1}^{d}{\left\|\mathrm{IP}\left(d_{i}\right)\right\|}^{2}\right)},$$

where $\gamma_{m}^{\prime }$ and $\gamma_{d}^{\prime }$ are the original bandwidths, and generally both are set to 1.

## 3 METHODS

**FIGURE 1:**
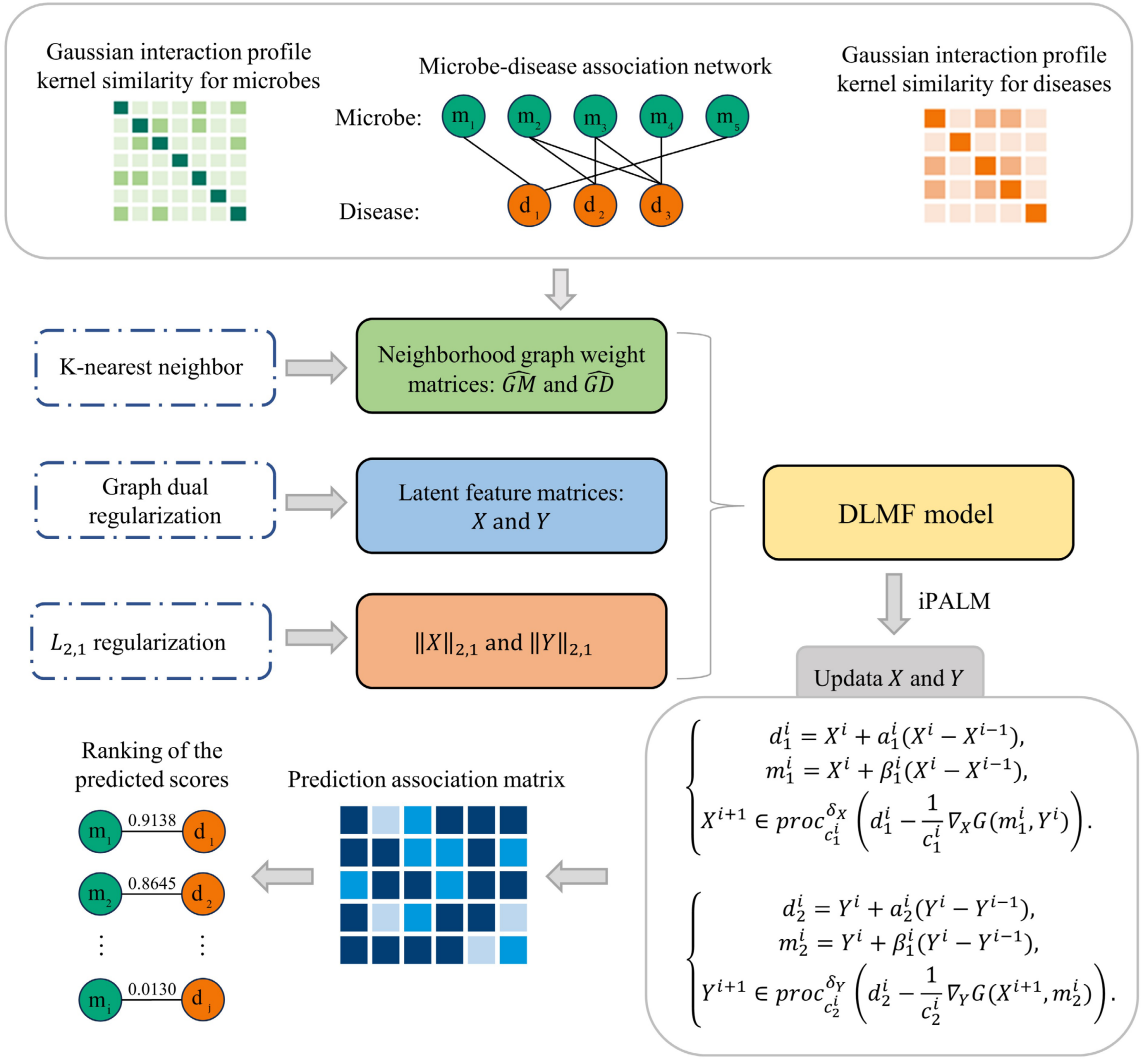
Flow chart of potential microbe–disease association prediction based on the computational model of iPALM‐GLMF.

In this paper, we presented a novel model iPALM‐GLMF, which modelled the microbe–disease associations prediction problem as a non‐negative factorization problem with graph dual regularization terms and $L_{2,1}$ norm regularization terms. iPALM‐GLMF took microbe–disease associations matrix A, Gaussian interaction profile kernel similarity of microbes GM and Gaussian interaction profile kernel similarity of diseases GD as inputs, and utilized GM and GD to construct the graph dual regularization terms, and solved the non‐negative matrix factorization problem of A using the graph dual regularization terms and $L_{2,1}$ norm regularization terms to obtain the feature matrices of microbes and diseases. Finally, the feature matrices were used to predict potential microbe–disease associations. A brief flow chart of the model iPALM‐GLMF is shown in Figure 1.

### 3.1 Non‐negative matrix factorization

The non‐negative matrix factorization (NMF) is a method for finding two low‐rank non‐negative matrices whose product approximates the original non‐negative matrix well.^40^ It incorporates non‐negative constraints to obtain a component‐based representation and enhances the interpretability of the problem accordingly. In microbe–disease associations prediction, the non‐negative matrix factorization (NMF) of associations matrices is widely used to obtain low‐dimensional feature representations of microbes and diseases in matrices space. The general form of NMF is as follows:

(5) $$\begin{array}{c} {\min\left\|A-XY^{T}\right\|}_{F}^{2}\\ s.t.X\geq 0,Y\geq 0. \end{array}$$

where X and Y represent the latent feature matrices of microbes and diseases, respectively. k is the rank of X and Y, $k\ll \min\left(m,d\right)$, $X\in {\mathbb{R}}^{m\times k}$, $Y\in {\mathbb{R}}^{d\times k}$. The non‐negativity constraint terms are adopted to ensure non‐negativity of X and Y.

### 3.2 Graph dual regularized non‐negative matrix factorization

Cai et al^41^ proposed a graph regularized non‐negative matrix factorization (GNMF) to find a compact representation that reveals hidden semantics while respecting the intrinsic geometric structure. It has been shown that learning performance can be greatly improved if the information about the flow structure contained in the data is utilized.^42^, ^43^ In addition, Shang et al.^44^ introduced graph dual regularization terms based on data manifolds and feature manifolds.

To obtain the geometric information of microbes and diseases, two *K*‐nearest neighbour graphs $N^{m}$ and $N^{d}$ are constructed for microbes and diseases based on GM and GD, respectively.

For two microbes $m_{i}$ and $m_{j}$, the weight of the edge between vertices i and j in graph $N^{m}$ is defined as follows.

(6) $$N_{\mathrm{ij}}^{m}=\left\{\begin{array}{c} 1,j\in N_{K}\left(i\right)\;\text{and}\;i\in N_{K}\left(j\right)\;\\ 0,j\notin N_{K}\left(i\right)\;\text{and}\;i\notin N_{K}\left(j\right)\\ 0.5,\text{otherwise}, \end{array}\right.$$

where $N_{K}\left(i\right)$ denotes the sets of K most similar microbes of microbes $m_{i}$ according to GM. Based on $N^{m}$ and GM, a sparse matrix ${\hat{\mathrm{GM}}}_{\mathrm{ij}}$ is computed as follows:

(7) $${\hat{\mathrm{GM}}}_{\mathrm{ij}}=N_{\mathrm{ij}}^{m}{\mathrm{GM}}_{\mathrm{ij}},\forall i,j.$$

Here $\hat{\mathrm{GM}}$ is a weight matrix representing the microbes neighbour graph. The graph Laplacian of $\hat{\mathrm{GM}}$ is $L_{m}=D^{m}-\hat{\mathrm{GM}}$, where $D^{m}$ is a diagonal degree matrix with $D_{\mathrm{ii}}^{m}=\sum_{r}{\hat{\mathrm{GM}}}_{\mathrm{ir}}$.

Similarly, the weight matrix $\hat{\mathrm{GD}}$ corresponding to the diseases neighbour graph is computed as follows:

(8) $${\hat{\mathrm{GD}}}_{\mathrm{ij}}=N_{\mathrm{ij}}^{d}{\mathrm{GD}}_{\mathrm{ij}},\forall i,j.$$

The graph Laplacian of $\hat{\mathrm{GD}}$ is $L_{d}=D^{d}-\hat{\mathrm{GD}}$, where $D^{d}$ is a diagonal degree matrix with $D_{\mathrm{jj}}^{d}=\sum_{q}{\hat{\mathrm{GD}}}_{\mathrm{jq}}$.

The normalized graph Laplacian forms of $L_{m}$ and $L_{m}$ are as follows:

(9) $${\tilde{L}}_{m}={\left(D^{m}\right)}^{-1/2}L_{m}{\left(D^{m}\right)}^{-1/2},$$

(10) $${\tilde{L}}_{d}={\left(D^{d}\right)}^{-1/2}L_{d}{\left(D^{d}\right)}^{-1/2}.$$

The optimization model of graph dual regularization terms non‐negative matrix factorization (GDNMF) of the microbe–disease interaction matrix A is formulated as follows:

(11) $$\begin{array}{c} \min_{\left(X,Y\right)}\frac{1}{2}{\left\|A-XY^{T}\right\|}_{F}^{2}+\lambda_{m}\mathrm{Tr}\left(X^{T}{\tilde{L}}_{m}X\right)+\lambda_{d}\mathrm{Tr}\left(Y^{T}{\tilde{L}}_{d}Y\right).\\ s.t.X\geq 0,Y\geq 0 \end{array}$$

where $\lambda_{m}$ and $\lambda_{d}$ are regularization parameters.

### 3.3 GDNMF with $L_{2,1}$ norm regularization terms

Zhang et al.^45^ introduced $L_{2,1}$ norm regularization terms into graph dual regularization non‐negative matrix factorization, which is used to ensure the sparsity of the matrices obtained by the factorization. The optimization model of GDNMF with $L_{2,1}$ norm regularization terms is formatted as follows:

(12) $$\begin{array}{c} \min_{\left(X,Y\right)}\frac{1}{2}{\left\|A-XY^{T}\right\|}_{F}^{2}+\lambda_{m}\mathrm{Tr}\left(X^{T}{\tilde{L}}_{m}X\right)+\lambda_{d}\mathrm{Tr}\left(Y^{T}{\tilde{L}}_{d}Y\right)+\lambda_{l}\left({\left\|X\right\|}_{2,1}+{\left\|Y\right\|}_{2,1}\right).\\ s.t.X\geq 0,Y\geq 0 \end{array}$$

where $\lambda_{l}$ is a regularization parameter, ${\left\|X\right\|}_{2,1}$ and ${\left\|Y\right\|}_{2,1}$ represent $L_{2,1}$ norms of matrix X and Y, respectively, and ${\left\|X\right\|}_{2,1}=\sum_{i}{\left(\sum_{j}{\left(x_{\mathrm{ij}}\right)}^{2}\right)}^{1/2}$, ${\left\|Y\right\|}_{2,1}=\sum_{i}{\left(\sum_{j}{\left(y_{\mathrm{ij}}\right)}^{2}\right)}^{1/2}$.

## 4 ALGORITHM

### 4.1 Non‐negative double singular value decomposition

Non‐negative double singular value decomposition (NNDSVD) is a method that improves the initialization phase of non‐negative matrix factorization (NMF) by providing valid and interpretable initial component matrices for matrix factorization.^35^ Based on the basic property of singular value decomposition, for matrix A, can be expressed as the sum of *k* leading singular factors $A=\sum_{i=1}^{k}\sigma_{i}u_{i}v_{i}^{T}$, where σ is the nonzero singular values of A, and ${\left\{u_{i},v_{i}\right\}}_{i=1}^{k}$ are the corresponding left and right singular vectors.

For a vector or matrix a, $a^{+}=\max\left(0,a\right)$ represents nonnegative section of a, $a^{-}=\max\left(0,-a\right)$ represents nonpositive section of a, $a=a^{+}-a^{-}$. $A=\sum_{i=1}^{k}\sigma_{i}u_{i}v_{i}^{T}$ can be transformed to the following form:

(13) $$A=\sum_{i=1}^{k}u_{i}v_{i}=\sum_{i=1}^{k}\left(u_{i}^{+}v_{i}^{+}+u_{i}^{-}v_{i}^{-}\right)-\left(u_{i}^{-}v_{i}^{+}+u_{i}^{+}v_{i}^{-}\right).$$

### 4.2 Proximal alternating linearized minimization

Bolte et al.^46^ introduced a Proximal Alternating Linearized Minimization method (PALM), which has global convergence results for nonconvex and nonsmooth semialgebraic problems.

Model (12) can be derived to the following form:

(14) $$\begin{array}{c} \min_{\left(X,Y\right)}\frac{1}{2}{\left\|A-XY^{T}\right\|}_{F}^{2}+R\left(X\right)+R\left(Y\right).\\ s.t.X\geq 0,Y\geq 0 \end{array}$$

where $R\left(X\right)=\lambda_{m}\mathrm{Tr}\left(X^{T}{\tilde{L}}_{m}X\right)+\lambda_{l}{\left\|X\right\|}_{2,1}$, $R\left(Y\right)=\lambda_{d}\mathrm{Tr}\left(Y^{T}{\tilde{L}}_{d}Y\right)+\lambda_{l}{\left\|Y\right\|}_{2,1}$. The nonnegative constraint of Formula (14) can be transformed to the following form:

(15) $$X\geq 0\to \delta_{X}=\left\{\begin{array}{c} X,X\geq 0,\\ \infty ,\text{otherwise}, \end{array}\right.$$

(16) $$Y\geq 0\to \delta_{Y}=\left\{\begin{array}{c} Y,Y\geq 0,\\ \infty ,\text{otherwise}. \end{array}\right.$$

Model (14) can be derived to the following form:

(17) $$\min\;\psi \left(X,Y\right)=\min\frac{1}{2}{\left\|A-XY^{T}\right\|}_{F}^{2}+R\left(X\right)+R\left(Y\right)+\delta_{X}+\delta_{Y}.$$

To solve model (17), the Gauss–Seidel method is used. The specific derivations are as follows:

(18) $$X^{i+1}\in \arg\min_{X}\psi \left(X,Y^{i}\right)$$

(19) $$Y^{i+1}\in \arg\min_{Y}\psi \left(X^{i+1},Y\right)$$

Let $G\left(X,Y\right)=\frac{1}{2}{\left\|A-\mathrm{XY}\right\|}_{F}^{2}+R\left(X\right)+R\left(Y\right)$, and substitute $Y^{i}$ into $\psi \left(X,Y\right)$ to remove the constant term and get $X^{i+1}\in \text{argmin}\left\{\delta_{X}+R\left(X\right)+{\left\|A-\mathrm{XY}\right\|}_{F}^{2}\right\}$, where $G\left(X,Y^{i}\right)$ is smooth function. Then the second‐order Taylor series of $G\left(X,Y^{i}\right)$ at a point $X^{i}$ is given by:

(20) $$X^{i+1}\in \arg\min_{X}\left\{\left\langle X-X^{i},\nabla_{X}G\left(X^{i},Y^{i}\right)\right\rangle +\frac{1}{2}\nabla_{X}\left(\nabla_{X}G\left(X^{i},Y^{i}\right)\right){\left\|X-X^{i}\right\|}_{F}^{2}+\delta_{X}\right\},$$

where $\nabla_{X}G$ is the partial derivative of G with respect to X.

Define the proximal map of f: ${\text{prox}}_{t}^{f}=\text{argmin}\left\{f\left(u\right)+\frac{1}{2t}{\left\|u-x\right\|}_{F}^{2},u\in {\mathbb{R}}^{m}\right\}$, where f: ${\mathbb{R}}^{m}\to \left(-\infty ,+\infty \right]$ is the lower semi‐continuous function to ensure non‐negativity, x is a fixed point, t is a constant. According to the definition of proximal map, the solution of Formula (20) is as follows:

(21) $$X^{i+1}\in {\text{prox}}_{c_{1}^{i}}^{\delta_{X}}\left(X^{i}-\frac{1}{c_{1}^{i}}\nabla_{X}G\left(X^{i},Y^{i}\right)\right).$$

where $c_{1}^{i}=\nabla_{X}\left(\nabla_{X}G\left(X^{i},Y^{i}\right)\right)={\left\|Y^{i}{\left(Y^{T}\right)}^{T}\right\|}_{F}$.

Then for a sequence ${\left(X^{i},Y^{i}\right)}_{i\in \mathbb{N}}$, parameters $c_{1}^{i}$ and $c_{2}^{i}$, we have

(22) $$\left\{\begin{array}{c} X^{i+1}\in {\text{prox}}_{c_{1}^{i}}^{\delta_{X}}\left(X^{i}-\frac{1}{c_{1}^{i}}\nabla_{X}G\left(X^{i},Y^{i}\right)\right),\\ Y^{i+1}\in {\text{prox}}_{c_{2}^{i}}^{\delta_{Y}}\left(Y^{i}-\frac{1}{c_{2}^{i}}\nabla_{Y}G\left(X^{i+1},Y^{i}\right)\right). \end{array}\right.$$

### 4.3 Inertial terms

Polyak has showed that the inertial term accelerates the convergence of the standard gradient method while the cost of each iteration remains essentially unchanged.^47^ A class of proximal methods has been considered by Attouch^48^ for maximal monotone operators in the context of second‐order differential equations in time. These methods are called the inertial proximal methods. In PALM, the commonly used optimization scheme is the first‐order gradient descent method, thus the inertial term is used in order to speed up the convergence.

### 4.4 Inertial proximal alternating linearized minimization

Let G denote the objective function of model (12). Then, model (12) can be expressed as follows:

(23) $$G\left(X,Y\right)=\frac{1}{2}{\left\|A-XY^{T}\right\|}_{F}^{2}+\lambda_{m}\mathrm{Tr}\left(X^{T}{\tilde{L}}_{m}X\right)+\lambda_{d}\mathrm{Tr}\left(Y^{T}{\tilde{L}}_{d}Y\right)+\lambda_{l}\left({\left\|X\right\|}_{2,1}+{\left\|Y\right\|}_{2,1}\right).$$

The partial derivatives of the function *G* with respect to *X* and *Y*, respectively, are as follows:

(24) $$\frac{\partial G}{\partial X}=\left(A-XY^{T}\right)Y^{T}+\lambda_{m}{\tilde{L}}_{m}X+\lambda_{l}\frac{\partial {\left\|X\right\|}_{2,1}}{\partial X}$$

(25) $$\frac{\partial G}{\partial Y}=\left(A-XY^{T}\right)Y^{T}+\lambda_{d}{\tilde{L}}_{d}Y+\lambda_{l}\frac{\partial {\left\|Y\right\|}_{2,1}}{\partial Y}$$

For sequences ${\left(X^{i},Y^{i}\right)}_{i\in \mathbb{N}}$, ${\left(m_{1}^{i},m_{2}^{i}\right)}_{i\in \mathbb{N}}$, ${\left(d_{1}^{i},d_{2}^{i}\right)}_{i\in \mathbb{N}}$, parameters $c_{1}^{i}$, $c_{2}^{i}$, $\beta_{1}^{i}$, $\beta_{2}^{i}$, we can get

(26) $$\left\{\begin{array}{c} \begin{array}{c} d_{1}^{i}=X^{i}+\alpha_{1}^{i}\left(X^{i}-X^{i-1}\right),\\ m_{1}^{i}=X^{i}+\beta_{1}^{i}\left(X^{i}-X^{i-1}\right), \end{array}\\ X^{i+1}\in {\text{prox}}_{c_{1}^{i}}^{\delta_{X}}\left(d_{1}^{i}-\frac{1}{c_{1}^{i}}\nabla_{X}G\left(m_{1}^{i},Y^{i}\right)\right). \end{array}\right.$$

(27) $$\left\{\begin{array}{c} \begin{array}{c} d_{2}^{i}=Y^{i}+\alpha_{2}^{i}\left(Y^{i}-Y^{i-1}\right),\\ m_{2}^{i}=Y^{i}+\beta_{2}^{i}\left(Y^{i}-Y^{i-1}\right), \end{array}\\ Y^{i+1}\in {\text{prox}}_{c_{2}^{i}}^{\delta_{Y}}\left(d_{2}^{i}-\frac{1}{c_{2}^{i}}\nabla_{Y}G\left(X^{i+1},m_{2}^{i}\right)\right). \end{array}\right.$$

**FIGURE 2:**
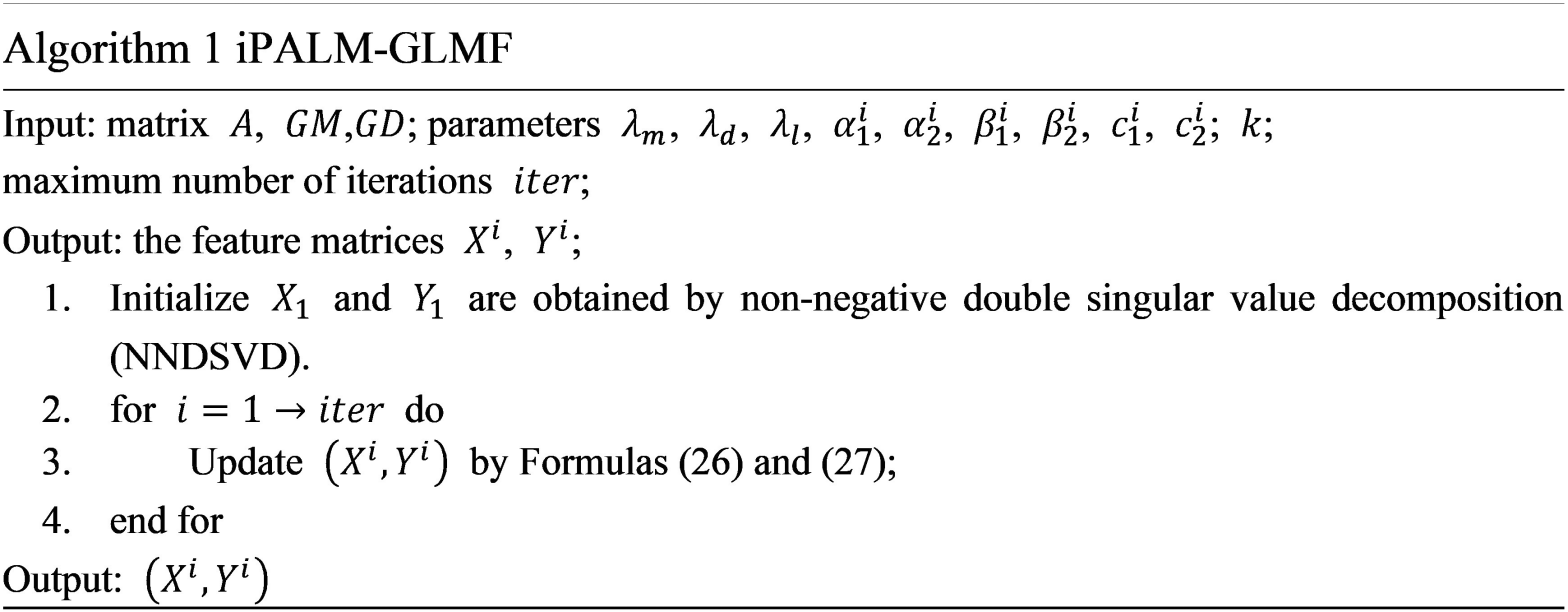
Pseudocode for the iPALM‐GLMF algorithm.

The detailed steps of iPALM‐GLMF are illustrated in Figure 2. The parameter values used in our model are set based on a previous study.^49^

## 5 EXPERIMENTS

### 5.1 Evaluation metrics

To evaluate the performance of iPALM‐GLMF, we performed two types of cross‐validations, namely global LOOCV and fivefold cross‐validation, in the datasets HMDAD and Disbiome. In global LOOCV, each time we took turns to select a sample from the recorded microbe–disease associations as a test sample and trained our model with the remaining known associations, and finally, the test sample was sorted with all unrecognized microbe–disease pairs. In fivefold cross‐validation, the known microbe–disease associations were randomly and uniformly divided into five parts, and each part was sequentially picked as a test sample, and the remaining four parts were used as training samples. As with global LOOCV, all unknown microbe–disease pairs were treated as candidate samples. To mitigate the possible impact of sample partitioning on the prediction effect, we randomly partitioned the known microbe–disease associations 100 times. In two cross‐validations, we implemented iPALM‐GLMF to obtain a list of scores for all microbe–disease pairs and ranked the scores of each test sample against the scores of the candidate samples. If the test sample ranked before a given threshold, we assumed that the model successfully predicted the association. Notably, we recalculated the microbe (disease) Gaussian interaction profile kernel similarity during each LOOCV and fivefold cross‐validation because the adjacency matrix changed when one or some of the known microbe–disease associations were removed.

### 5.2 Performance comparison

Based on the cross‐validation results, we used AUPR and AUC values as metrics to evaluate the performance of iPALM‐GLFM. We compared iPALM‐GLFM with the following state‐of‐the‐art methods on the same dataset.

KATZHMDA^15^ based on KATZ measure achieves the prediction of potential disease–microbe association through calculating the number and length of paths between two nodes in microbe–disease heterogeneous network.

LRLSHMDA^50^ is a semi‐supervised learning calculation model based on Laplacian regularized least squares classification.

NTSHMDA^51^ is a random‐walk based predictive model which predicts human microbe–disease associations by integrating network topological similarities.

BiRWHMDA^52^ is a method for predicting potential microbe–disease associations by double random walks on heterogeneous networks.

ABHMDA^29^ is a model for revealing disease‐associated microbes through a strong classifier composed of weak classifiers with corresponding weights.

**FIGURE 3:**
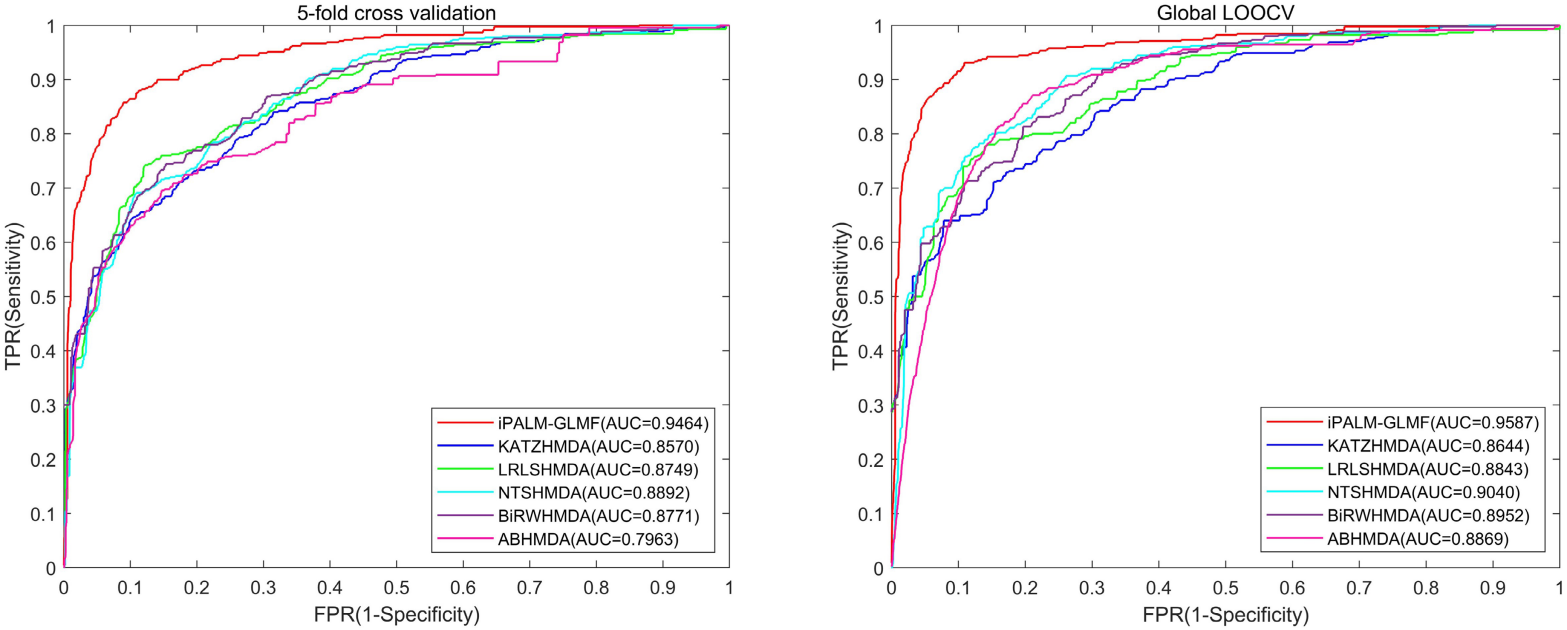
The graphs show the AUCs of iPALM‐GLMF in fivefold cross‐validation (0.9464) and global LOOCV (0.9587) under the HMDAD database, respectively, which outperformed all the aforementioned models (KATZHMDA, LRLSHMDA, NTSHMDA, BiRWHMDA and ABHMDA).

**FIGURE 4:**
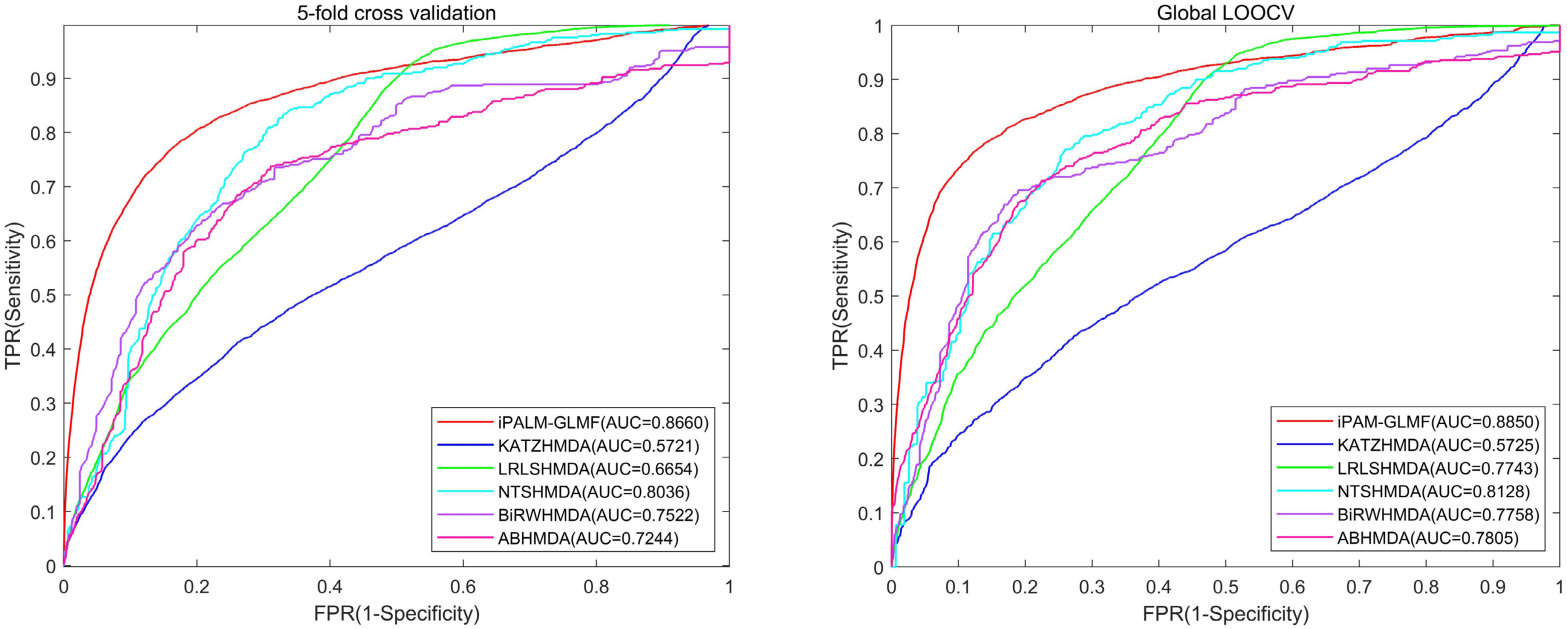
The graphs show the AUCs of iPALM‐GLMF in fivefold cross‐validation (0.8660) and global LOOCV (0.8850) under the Disbiome database, respectively, which outperformed all the aforementioned models (KATZHMDA, LRLSHMDA, NTSHMDA, BiRWHMDA and ABHMDA).

Our method was compared with five baselines under fivefold cross‐validation and global LOOCV on two datasets, namely HMDAD and Disbiome. We used AUC values and AUPR values as indicators to evaluate each method. For better visual comparison, the corresponding ROC curves for iPALM‐GLMF, KATZHMDA, LRLSHMDA, NTSHMDA, BiRWHMDA and ABHMDA were shown in Figures 3 and 4.

On the HMDAD database, iPALM‐GLMF performed best compared to the other five baseline methods, with average AUCs of 0.9464 ± 0.0039 and 0.9587 under fivefold cross‐validation and global LOOCV, respectively. On the Disbiome database, iPALM‐GLMF performed best compared to the other five baseline methods, with average AUCs of 0.8660 ± 0.0015 and 0.8850 under fivefold cross‐validation and global LOOCV, respectively. The results indicated that our method was effective in predicting novel microbe–disease associations.

**FIGURE 5:**
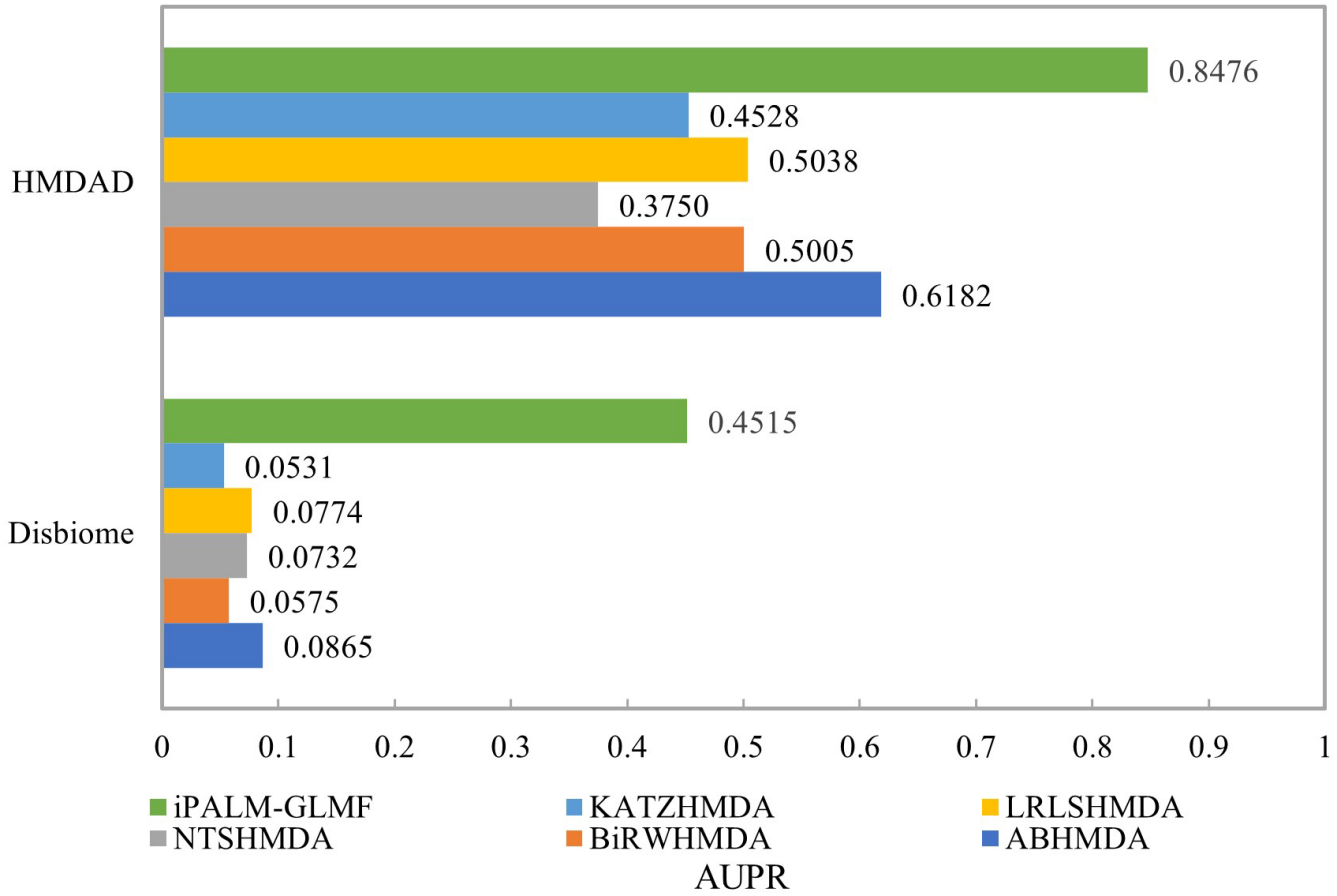
Comparison of AUPR values for iPALM‐GLMF and five other methods using fivefold cross‐validation.

To further evaluate the validity of our model, the AUPR values under fivefold cross‐validation for the two databases were shown in Figure 5. The average AUPR of our method under HMDAD and Disbiome databases were: 0.8476 and 0.4515, respectively, which were better than the baseline method.

The performance of iPALM‐GLMF on HMDAD, Disbiome at fivefold cross‐validation was summarized in Table 2. It could be seen that our method had the best AUC and AUPR on the HMDAD dataset. The main reason may be that the Disbiome was sparser than the HMDAD. The density of HMDAD was 3.95% and the density of Disbiome was 1.90%. Therefore, the iPALM‐GLMF method was better trained on HMDAD than Disbiome.

**TABLE 2:** Performance of the iPALM‐GLMF method under fivefold cross‐validation on two datasets.

Dataset	AUC	AUPR
HMDAD	0.9464 ± 0.0039	0.8476
Disbiome	0.8660 ± 0.0015	0.4515

### 5.3 Ablation experiment

In this section, we sought to determine the impact of several techniques on the performance of our proposed iPALM‐GLMF. To this end, we evaluated iPALM‐GLMF, iPALM‐GLMF (without NNDSVD, i.e. SVD was used in the initialization phase of the matrix factorization), iPALM‐GLMF ($\lambda_{m}=0$, i.e. the graph regularization term for microbe was not used), iPALM‐GLMF ($\lambda_{d}=0$, i.e. the graph regularization term for disease was not used), iPALM‐GLMF ($\lambda_{l}=0$, i.e. $L_{2,1}$ norm regularization term was not used) and PALM‐GRMF (i.e. inertial forces was not used). The results of the above settings were shown in Tables 3 and 4. In fivefold cross‐validation, the iPALM‐GLMF showed better performance than in other settings.

**TABLE 3:** AUC values of different algorithms under fivefold cross‐validation.

Method	HMDAD	Disbiome
iPALM‐GLMF	**0.9464 (0.0044)**	**0.8660 (0.0064)**
iPALM‐GLMF (without NNDSVD)	0.8170 (0.0020)	0.8353 (0.0007)
iPALM‐GLMF ($\lambda_{m}=0$)	0.9190 (0.0040)	0.8410 (0.0025)
iPALM‐GLMF ($\lambda_{d}=0$)	0.9323 (0.0059)	0.8539 (0.0014)
iPALM‐GLMF ($\lambda_{l}=0$)	0.9421 (0.0039)	0.8594 (0.0016)
PALM‐GLMF	0.9412 (0.0017)	0.8557 (0.0023)

*Note*: The maximum AUC on each dataset is shown in bold. Standard deviation is shown in parentheses.

**TABLE 4:** AUPR values of different algorithms under fivefold cross‐validation.

Method	HMDAD	Disbiome
iPALM‐GLMF	**0.8584 (0.0046)**	**0.4520 (0.0059)**
iPALM‐GLMF (without NNDSVD)	0.5065 (0.0042)	0.2544 (0.0028)
iPALM‐GLMF ($\lambda_{m}=0$)	0.8214 (0.0062)	0.4205 (0.0024)
iPALM‐GLMF ($\lambda_{d}=0$)	0.8368 (0.0074)	0.4311 (0.0041)
iPALM‐GLMF ($\lambda_{l}=0$)	0.8565 (0.0048)	0.4519 (0.0025)
PALM‐GLMF	0.8574 (0.0051)	0.4513 (0.0061)

*Note*: The maximum AUPR on each dataset is shown in bold. Standard deviation is shown in parentheses.

### 5.4 Case studies

To further evaluate whether iPALM‐GLMF could demonstrate accurate and robust performance, we conducted case studies on two different types of diseases under colorectal cancer, inflammatory bowel disease (IBD) and asthma. These studies were conducted using the HMDAD database.

In the first case study, we performed potential microbe prediction for colorectal cancer and Inflammatory bowel disease (IBD). Specifically, we categorized all unknown samples under the same disease and verified whether the association between the top 10 microbes and the disease under study was validated by relevant literature. Colorectal cancer is the second leading cause of cancer deaths in the United States, and the incidence in young adults is increasing each year.^53^ Therefore, there is an urgent need for novel and sensitive biomarkers that can detect colorectal cancer in an effective and timely manner. Researchers have linked many microbes to colon cancer. For example, D. A. Geier and M. R. Geier^54^ unearthed a potential link between *Clostridium difficile* intestinalis infection and colon cancer incidence, finding that adults with *Clostridium difficile* intestinalis had a significantly increased incidence of colon cancer. Another example is the discovery by Ralser et al.^55^ that *Helicobacter pylori* promotes colorectal carcinogenesis by deregulating intestinal immunity and inducing a mucus‐degrading microbiota signature, based on the evidence they provide suggesting that *H. pylori* infection is a strong causal facilitator of colorectal carcinogenesis. As shown in Table 5, we implemented iPALM‐GLMF to discover potentially relevant microbe for colorectal cancer and found that 9 out of the top 10 predictions were confirmed by relevant literature.

**TABLE 5:** The top 10 potential microbes related to colorectal cancer identified by iPALM‐GLMF.

Rank	Microbe	Evidence
1	*Helicobacter pylori*	PMID: 38328335
2	*Clostridium difficile*	PMID: 38193707
3	Clostridium coccoides	PMID: 28661219
4	Clostridia	PMID: 38068869
5	Lachnospiraceae	PMID: 28988196
6	Desulfovibrio	Unconfirmed
7	Prevotellaceae	PMID: 37469407
8	Bacteroidetes	PMID: 29170280
9	Porphyromonadaceae	PMID: 37072632
10	*Bacteroides vulgatus*	PMID: 38033588

The main types of inflammatory bowel disease (IBD), which include Crohn's disease and ulcerative colitis, are caused in part by bacteria that may activate the patient's immune system to attack foreign bodies.^56^ Once activated, the patient's immune system has difficulty regulating and destroying the gastrointestinal tract, leading to IBD symptoms. Recent research indicates a close correlation between various microorganisms and IBD. For instance, a reduction in members of the phyla Bacteroidetes and Firmicutes has been observed in IBD, particularly across different variants.^57^ As shown in Table 6, we implemented iPALM‐GLMF to discover potentially relevant microbe for IBD and found that 10 of the top 10 predictions were confirmed by relevant literature. The high prediction accuracy suggested that our model could be used for real‐life applications.

**TABLE 6:** The top 10 potential microbes related to inflammatory bowel disease identified by iPALM‐GLMF.

Rank	Microbe	Evidence
1	*Clostridium* difficile	PMID: 37894185
2	Bacteroidetes	PMID: 37894185
3	*Desulfovibrio*	PMID: 29462845
4	Firmicutes	PMID: 25307765
5	*Staphylococcus epidermidis*	PMID: 33618750
6	Clostridium coccoides	PMID: 19235886
7	Clostridia	PMID: 25307765
8	*Staphylococcus*	PMID: 33618750
9	Enterobacteriaceae	PMID: 30319571
10	Clostridiales	PMID: 38335423

**FIGURE 6:**
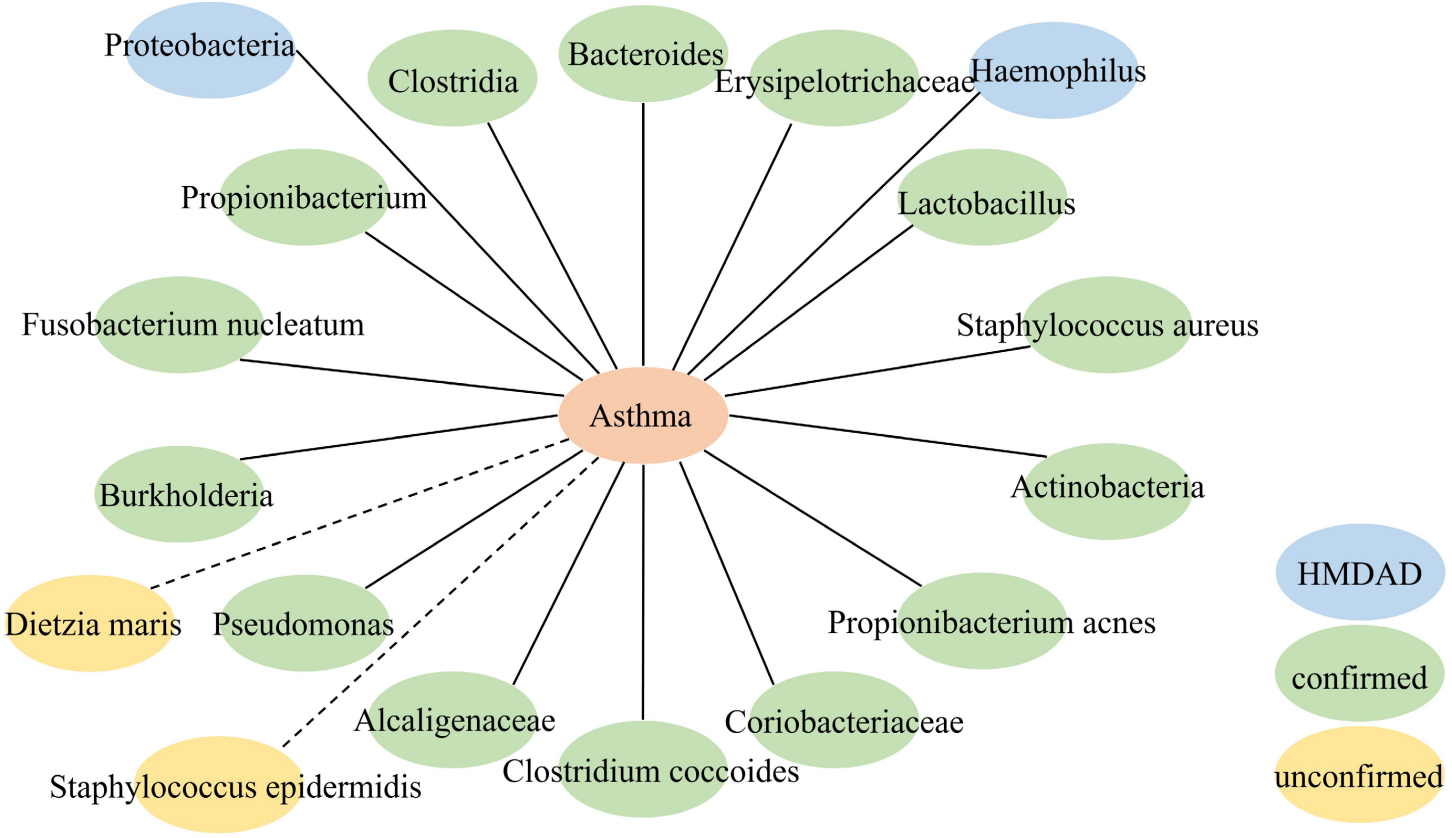
Prediction results of top‐20 Asthma‐associated microbes.

In the second case study, we performed relevant microbe prediction for asthma with the goal of evaluating the model's ability to predict associations between unknown microbes and disease in the absence of any known relevant microbes. Specifically, we replaced all microbes associated with a specific disease in the adjacency matrix with zero. After model prediction, we validated the number of microbes sampled in the top 20 ranked diseases confirmed in the relevant literature, as shown in Figure 6, with results demonstrating that HMDAD has included 2 microbes as well as 16 microbes that have been confirmed in the literature, with only 2 that have not yet been validated. In other words, 18 of the top 20 microbes predicted by our model have been confirmed, further demonstrating the validity of iPALM‐GLMF.

## 6 CONCLUSION

Recognizing potential microbe–disease associations not only contributes to disease diagnosis, treatment and prognosis, but also to microbe‐oriented therapies in precision medicine. In this article, we proposed a novel matrix factorization‐based model called iPALM‐GLMF for inferring potential microbe–disease associations. In the model, we combined graph dual regularization terms with $L_{2,1}$ norm regularization terms, which was used to capture information about the geometric structure in the microbe similarity matrix and the disease similarity matrix and to ensure the sparsity of the matrices obtained from the nonnegative matrix factorization. We then solved the matrix factorizations with graph dual regularization terms and $L_{2,1}$ norm regularization terms using an inertial proximal alternating linearization minimization algorithm that achieves global convergence. Cross‐validation results demonstrated the superior performance of iPALM‐GLMF over many previous computational methods. Case studies on colorectal cancer, inflammatory bowel disease and asthma also showed that our model could exhibit reliable and accurate predictive performance. It is reasonable to conclude that iPALM‐GLMF will be useful for microbe–disease association prediction.

Although our model showed good performance, there are still some limitations and further improvements are needed in the future. Fewer microbe–disease pairs have been confirmed to be associated, and the similarity information about diseases and microbes is not diverse enough, which seriously affects the predictive performance of the model. In future work, we will collect more correlation as well as similarity information and combine the advantages of the existing models to construct models with stronger predictive ability. In addition, considering the high application value of genetic information, the introduction of host genetic information is bound to greatly increase the prediction performance of the model.^58^, ^59^, ^60^ Moreover, after more and more potential microbe–disease associations have been predicted and confirmed, we can further predict microbe–drug associations based on microbe–disease association information and other related data, which is conducive to providing new strategies for drug design and disease treatment.

## AUTHOR CONTRIBUTIONS

**Ziwei Chen:** Conceptualization (equal); methodology (equal); supervision (equal); writing – review and editing (equal). **Liangzhe Zhang:** Investigation (equal); methodology (equal); validation (equal); visualization (equal); writing – original draft (equal). **Jingyi Li:** Formal analysis (equal); methodology (equal); software (equal); validation (equal). **Hang Chen:** Supervision (equal); writing – review and editing (equal).

## CONFLICT OF INTEREST STATEMENT

The authors declare that the research was conducted in the absence of any commercial or financial relationships that could be construed as a potential conflict of interest.

## Data Availability

The original contribution from the study is included in the article, further inquiries can be directed to the corresponding author.
